# Supplementary material for: Sex Differences in Anderson–Fabry Cardiomyopathy: Clinical, Genetic, and Imaging Analysis in Women
Source: Genes (Basel). 2023 Sep 15;14(9):1804. doi: 10.3390/genes14091804 (PMC10531426; doi:10.3390/genes14091804)
Supplement: Supplementary file 1 [file genes-14-01804-s001.zip › genes-2592602-Supplemental material.pdf]

Supplemental material

Table S1. Prevalence of Variants of conflicting interpretation in the entire population who underwent genetic screening in the timeframe (N=173- PANEL A), and in the study population (N=72- Panel B).

Panel A	type	variant	tot	N° families	Males	Females
Conflicting interpretation*	VUS/likely benign/benign variants	**S126G**	27 (18.7%)	8	10	17
		**A143T**	26 (18%)	2	6	20
		**D313Y**	13 (9%)	4	4	9

Panel B	type	variant	N° tot	AFD-LVH	AFD-N	Males	Females
Conflicting interpretation*	VUS/likely benign/benign variants	**S126G**	13 (18%)	1 F	12	3 (15.8%)	10 (18.9%)
		**A143T**	8 (11.1%)	-	8	3 (15.8%)	5 (9.4%)
		**D313Y**	8 (11.1%)	2 F	6	2 (10.5%)	6 (11.3%)

*We grouped these three variants under a single category called "of conflicting interpretation of pathogenicity", as reported in the ClinVar portal because their interpretation is still debated between VUS and. Refer to https://www.ncbi.nlm.nih.gov/clinvar/- see in the text.

Table S2. Comparison between patients undergoind therapy (N=26), divided in two subgroups (ERT and Migalastat), and patients not undergoing any therapy (N=46) - General characteristics.

	Therapy N=26	ERT (N=18)	Migalastat (N=8)	No therapy (N=46)
**Age, yo**	47.1±16.2	48.3±15.6	44.5±18.4	43.7 ± 16.1
**M**	12(46.2%)	6 (33.3%)	6(75%)	7(15.2%)
**F**	14(53.8%)	12 (66.7%)	2(25%)	39(84.8%)
**Hypertension**	12(46.2%)	10 (55.6%)	2(25%)	12 (26.1%)
**Diabetes**	2(7.7%)	2(11.1%)	0	7 (15.2%)
**Smoke**	4(15.4%)	4(22.2%)	0	10 (21.7%)
**Dyslipidemia**	4 (15.4%)	3(16.7%)	1 (12.5%)	5 (10.9%)
**CKD**	5 (19.2%)	4(22.2%)	1 (12.5%)	4 (8.7%)
**Stroke/TIA**	4(15.4%)	3 (16%)	1(12.5%)	2 (4.3%)
**Syncope**	2(7.7%)	1 (5.6%)	1 (12.5%)	4 (8.7%)
**Fam.hystory SCD**	0	0	0	2 (2.2%)
**Fam.hystory AFD**	17(65.4%)	11(61.1%)	6 (75%)	24 (52.2%
**Kidney transpl.**	6(8%)	4(30,8%)	2(3,2%)	0
**α-Gal A (nmol/ml/h)**	3.7 [0.5-8.9]	3.9 [0,9-8.7]	3.5 [0.5-10.2]	5.7 [2.8-12.4]
**Lyso GB3 baseline (nmol/l)***	2.7 [1.5-9.4]	6.8 [1.5-28]	1.9 [1.4-6.2]	1.5 [1.1-1.6]
**CV Death**	2 (2,8%)	2(11.1%)	0	0
**NYHA 1**	16(61.5%)	10(55.6%)	6 (75%)	37 (80.4%)
**NYHA 2**	9(34.7%)	7(38.9%)	2 (25%)	9 (19.6%)
**NYHA 3**	1(3.8%)	1(5.6%)	0	0
**BP-sys(mmHg)**	127.5 [120-140]	127.5 [120-140]	127.5 [120-142.5]	122.5 [115-130]
**BP-dia(mmHg)**	77.5 [70-81.2]	77.5 [72.5-80]	75 [70-88.7]	80 [70-80]
**HR (bpm)**	68.6±9.7	68.9±10.2	68.1±9	70±8.3
**ICD**	1(3.8%)	1(5.6%)	0	0
**PM**	1(3.8%)	0	1 (12.5%)	0
**GLA variant :**				
• **Classic**	10 (38.5%)	9(50%)	1(12.5%)	3 (6.5%)
• **Late-onset**	8 (30.8%)	3 (16.7%)	5 (62.5%)	22 (47.8%%)
• **VUS**	8 (30.8%)	6 (33.3%)	2 (25%)	21 (45.7%)

NB: During follow up one patients switched from migalastat to ERT, and conversely another one switched from ERT to migalastat. *for Lyso-GB3 baseline values: significant difference for all groups (P 0.006) and for therapy vs no-therapy (P 0.002). For abbreviations: see previous tables.

Table S3. Comparison between patients undergoing therapy (N=26), divided in two subgroups (ERT and Migalastat), and patients not undergoing any therapy (N=46) – Instrumental exams findings.

	Therapy N=26	ERT N=18	Migalastat N=8	No therapy N=46
**RBBB**	1(3.8%)	1(5.6%)	0	0
**AVB-III**	1(3.8%)	0	1 (12.5%)	0
**Short PR**	3(11.5%)	3(16.7%)	0	0
**Holter ECG done**	9(34.6%)	8(44.4%)	1(12.5%)	3(6,5%)
**VT/NSVT**	4(15.4%)	3 (16.7%)	1(12.5%)	2(4.3%)
**AF**	5(19.2%)	4(22.2%)	1 (12.5%)	2(4.3%)
**CMR done**	14(53.8%)	11 (61.1%)	3(37.5%)	0
**LGE+ (% tot)**	9(34.6%)	7 (38.9%)	2(25%)	0
**LGE+ (% pts with CMR)**	9/14 (64,2%)	7/11 (63.6%)	2/3 (66.6%)	0
**N° segments LGE**	0 [0-2]	0.5 [0-2]	2 [0-5]	0

Table S4. Comparison between patients undergoing therapy (N=26), divided in two subgroups (ERT and Migalastat), and patients not undergoing any therapy (N=46) – Echo parameters.

	Therapy N=26	ERT N=18	Migalastat N=8	No therapy N=46	P ERT vs Migalas-tat vs NO th.	P therapy vs no th.	P ERT vs Migalastat
**IVS (mm)**	9 [7.4-11.4]	9 [7-12.6]	9 [7.9-9.7]	8 [6.4-9]	0.09	**0.03**	0.72
**PW (mm)**	8.9 [7.5-11.7]	10 [7.9-12]	8 [7-8.9]	8 [7-9]	**0.02**	**0.02**	0.16
**LVMi (g/sqm)**	80 [65.7-111.5]	88 [68.7-116]	69 [64.5-81.5]	67.5[52.7-79]	**0.006**	**0.003**	0.24
**EF, %**	65 [61.7-68.5]	66 [63.2-70.2]	64.5 [59.7-67.2]	65[61.7-67.2]	0.60	0.85	0.31
**LAVi (ml/sqm)**	25.5 [21-37.2]	26.5 [21-42.2]	24 [17.7-35.5]	22.5[17-28]	0.15	0.08	0.40
**E/e'**	8 [5-10]	9 [5.7-13]	7.5 [4.2-8.7]	7[6-10]	0.40	0.87	0.16
**TR-Vmax (m/s)**	2.3 [1.7-2.4]	2.3 [1.7-2.4]	2 [1.5-2.2]	2.3[2-2.4]	0.25	0.56	0.16
**LV-GLS (%)**	-17.5 [-12.5/-20.2]	-17 [-11/ -20.2]	-18.5 [-16.2/-21.2]	-19 [-17/-21.5]	0.18	0.09	0.57
**LV-MD (ms)**	37.5 [28-72.7]	37.5 [28.7-87]	35.5 [27.2-64.2]	40[31-49.5]	0.86	0.89	0.57

NB: During follow up one patients switched from migalastat to ERT, and conversely another one switched from ERT to migalastat. For abbreviations: see previous tables.

Table S5. comparison between females undergoing therapy and males undergoing therapy.

	F therapy (N=14)	M therapy (N=12)	P-value
**AGE**	53.3±14.8	38,8 ± 14,6	
**CLASSIC variant**	6 (42.9%)	4 (21%)	
**LATE-ONSET**	3 (21.4%)	7 (36,9%)	
**VUS**	5 (35.7%)	8 (42,1%)	
**α-Gal A (nmol/ml/h)**	8.2 [5.1-10.4]	1.8 [0.3-3.35]	**0.003**
**LYSO-GB3 (nmol/l)**	3.7 [1.2-7.6]	1.9 [1.5-17.3]	0.28
**V-AR**	2 (14.3%)	2 (10,5%)	
**AF**	3 (21.4%)	2 (10,5%)	
**Pts with LVH**	4 (28.6%)	3 (15,8%)	

**LVMi (g/sqm)**	75.5 [63.5-111.5]	78 [73-96]	0.63
**EF, %**	65.5 [64-70.2]	64 [60-68]	0.46
**E/e′**	9 [7.5-13]	7 [5-10]	0.06
**LV-GLS, %**	-18.5 [-12.5/-22.2]	-17 [-14/-20]	0.40
**MD (ms)**	37.5 [28.7-72.7]	41 [29-60]	0.94
**LGE+, n(%)**	4/7 [57% of CRM]	6/7 [85.7% of CMR]	
**LGE segments**	0 [0-3]	2 [2-5]	0.66

For abbreviations: see previous tables.

Table S6. Among females, comparison between patients undergoing therapy (N=4 of which 13 ERT and only 1 Migalastat) and patients not undergoing therapy.

	**AFD-Females TH (N=14)**	**AFD- Females NO th (N=39)**	**P value**
**Age (yo)**	62,5 ± 3,9	44.9 ± 16.3	
**α-Gal A (nmol/ml/h)**	8.2 [5.1-10.4]	6.7 [4.5-13.4]	0.78
**Lyso Gb3 (nmol/l)**	3.7 [1.2-7.6]	1.4 [1.1-1.6]	0.06
**Classic variant**	6 (42.9%)	3 (7.7%)	
**Late onset**	3 (21.4%)	19 (48.7%)	
**VUS**	5 (35.7%)	16 (41%)	
**V-AR**	2 (14.3%)	2 (5.1%)	
**AF**	3 (21.4%)	2 (5.1%)	
**LVMi (g/sqm)**	75.5 [63.5-111.5]	62 [52-82]	**0.02**
**EF, %**	65.5 [64-70.2]	65 [62-67]	0.74
**E/e′**	9 [7.5-13]	7 [6-10]	0.13
**GLS, %**	-18.5 [-12.5/-22.2]	-19.5 [-17/-22]	0.35
**MD, msec**	37.5 [28.7-72.7]	37 [30.7-49.2]	0.98

For abbreviations: see previous tables.

Table S7. Among males, comparison between patients undergoing therapy (N=12, of which 6 ERT and 6 Migalastat) and patients not undergoing therapy.

	**AFD-males TH (N=12)**	**AFD-males NO th (N=7)**	**P value**
**Age (yo)**	42.5 ± 15.3	36.8 ± 14	
**α-Gal A (nmol/ml/h)**	8,5 [4,8-10,9]	2.8 [1.7-5.7]	0.16
**Lyso Gb3 (nmol/l)**	0.5 [0.3-3]	1.6 [1.3-1.6]	0.08
**Classic mutation**	4 (33.3%)	0	
**Late onset**	5 (41.7%)	2 (28.6%)	
**VUS**	3 (25%)	5 (71.4%)	
**V-AR**	2 (16.7%)	0	
**AF**	2 (16.7%)	0	

**LVMi (g/sqm)**	80 [70.2-111]	74 [73-78]	0.20
**EF, %**	64.5 [59.7-67.7]	64 [60-69]	0.90
**E/e'**	6.5 [4.2-9.5]	7 [6-11]	0.30
**GLS, %**	-17 [-11.5/-19.7]	-17 [-14/-20]	0.77
**MD, msec**	44.5 [25.7-82.2]	41 [31-60]	0.90

For abbreviations: see previous tables.

Table S8. Among females with AFD-LVH, comparison between patients undergoing therapy (all ERT) and patients not undergoing therapy.

	**AFD-LVH Females therapy (N=4)**	**AFD-LVH Females No therapy (N=5)**	**P value**
**Age (yo)**	60.2 ± 3.0	64.4 ± 3.7	
**α-Gal A (nmol/ml/h)**	8.2 [3.2-10.3]	9.2 [4.8-9.2]	0.69
**Lyso Gb3 (nmol/l)**	8.7 [8-8.7]	5.1 [1,1-5.1]	0.66
**Classic variant**	4 (100%)	0	
**Late onset**	0	2 (40%)	
**VUS**	0	3 (60%)	
**V-AR**	2 (50%)	1 (20%)	
**AF**	3 (75%)	2 (40%)	
**LVMi (g/sqm)**	137.5 [104.7-180.7]	104 [101.5-118]	0.29
**EF, %**	65.5 [58-73]	65 [63.5-71]	0.73
**LAVi, ml/sqm**	48.5 [32.5-60]	34 [29-49]	0.41
**E/e'**	13 [7.7-16]	10 [7.5-15]	0.73
**TR V-max (m/s)**	2.3 [1.4-2.4]	2.4 [2.3-2.8]	0.19
**GLS, %**	-10 [-5.2/-12.5]	-18 [-12.3/-20]	0.06
**MD, msec**	90.5 [43.5-196.7]	66 [48-74]	0.29

For abbreviations: see previous tables.

Table S9. Among females and males with AFD-N, comparison between patients undergoing therapy and patients not undergoing therapy.

	**AFD-N Females therapy N=10**	**AFD-N Females NO therapy N=34**	**P value**	**AFD-N therapy Males N=9**	**AFD-N no therapy Males N=7**	**P value**
**Age, yo**	50.5 ± 16.8	42.1 ± 15.5		34.9 ± 12.8	36.8 ±14	
**LVMi (g/sqm)**	68 [59-84.5]	59 [51.7-72.7]	0.08	79 [66-81]	74 [73-78]	0.60
**EF%**	65.5 [65-67.2]	65.5 [61.7-67.2]	0.71	64 [59-69]	64 [60-69]	1
**LAVi (ml/sqm)**	22.5 [21-28]	21.5 [16-27.2]	0.31	24 [14.5-28.5]	25 [17-26]	1
**E/e'**	9 [7.2-9.2]	7 [6-9.2]	0.29	5 [4-7]	7 [6-11]	0.05
**TR-Vmax(m/s)**	2 [1.5-2.7]	2.3 [1.9-2.4]	0.65	2 [1.7-2.4]	2 [1.8-2.1]	0.30
**LV-GLS (-%)**	-20.5 [-17.7/-23]	-20 [-17/-22]	0.68	-18 [-16.5/-20]	-17 [-14/-20]	0.54

**LV-MD (ms)**	32.5 [27.7-41.2]	35 [30.5-47]	0.47	29 [24-60.5]	41 [31-60]	0.30

For abbreviations: see previous tables.

Table S10. (supplement) - Patients undergoing therapy (ERT or migalastat)- comparison of pre-treatment parameters and in-treatment follow up (at 1 year and at the longest available follow up).

	**Baseline (N=6 ERT and 5 migalastat)**	**FU at 1 year**	**Longest available FU (median 4 years)**	**P baseline vs 1-y FU**	**P baseline vs longest FU**
**Age (yo)**	44.8 ± 16.4	/	/	/	/
**α-Gal A (nmol/ml/h)**	2.7 [0.3-8.5]	8.6 [2.0-15]	15 [0.2-15]	0.17	0.37
**Lyso-Gb3 (nmol/l)**	9.4 [2.0-40.4]	6.1 [1.5-13.9]	5 [1.9-39]	0.35	0.68
**V-AR, n (%)**	2 (18.2%)	2 (18.2%)	0		
**AF, n (%)**	1 (9.1%)	2 (18.2%)	0		
**NYHA 1, n (%)**	8 (72.7%)	9 (81.8%)	11 (100%)		
**NYHA 2, n (%)**	3 (27,3%)	2 (18.2%)	0		
**LVH, n (%)**	3 (27.3%)	3 (27.3%)	3 (27.3%)		
**IVS, mm**	9 [7-13]	9 [6.75-11.5]	10 [8-14]	0.86	0.42
**LWP, mm**	8 [7-11]	8 [6.7-11]	9 [7-10.5]	0.65	0.52
**LVMi, g/sqm)**	69 [62.7-89]	76.5 [64-99.7]	86 [66-120.2]	0.72	0.37
**EF, %**	65 [62-66]	65 [63.7-66]	64 [62-65]	0.81	0.54
**E/e'**	6.3 [5-8.7]	8.2 [4.6-11.2]	7.9 [6.6-10.0]	0.42	0.11
**LAVi, ml/sqm**	26 [18.5-37.5]	28.8 [18.6-38.5]	26.5 [18.2-39]	0.86	0.95
**TRVmax**	2 [1.3-2.2]	1.8 [1.2-2.3]	2.2 [2.1-2.5]	0.91	0.12
**GLS, %**	-19.5 [-10/-18,5]	-19 [-15/-23]	-20 [-17.25/-22.5]	0.65	0.49
**MD, ms**	45 [27.2-82.2]	36.5 [28.5-57]	35.5 [24.7-69.5]	0.70	0.75

Only patients for which at least one control visit with advanced echo and biochemical markers was available have been included (11 patients, of which 6 on ERT and 5 on migalastat). There have been two crossing-over (1 patient from ERT switched to migalastat, and another patient viceversa. For abbreviations: see previous tables.

Table S11. Comparison with hystorical cohorts from literature in AFD patients (without sex discrimination).

	**Our total AFD cohort, N=72**	**FOS (Kampmann, 2015)[1] N=45**	**FOS (Ramaswami, 2019)[2] N=69**	**FAMOUS cohort (Lenders, 2020)[3] N=59**	**El Sayed, 2021 [4] N=213**	**Avanesov, 2023 [5] (classical cohort) N=37***
**Age first visit (yo)**	45 ± 16.1	34.7 ±12.8		49±13	50 [19-83]	42 ± 11
**Age ERT start**		34.7 ± 12.8	38.80 (3.7–67.3)		42 [10-77]	
**α-Gal A (nmol/ml/h)**	5.0 [2.6-9.4]					
**Lyso Gb3 (nmol/l)**	1.5 [1.3-2.7]					16±16 ng/ml
**hypertension**	33.3%	11.1			24%	

**diabetes**	12.5%	0		11.5%	2%	
**BP-sys**	125 [120-135]	126.8 14.4	123.1±14.4			
**BP-dia**	80 [70-80]	68.2± 10.3	72.8±11.2			
**smoke**	19.4%	11.1%		11.5%	42%	
**SCD**	1.4%				**9 (4%)	
**Conduction abnormalities**	11.1%					
**PM/ICD**	1.4%/1.4%			1.7%/13.6%		
**V-AR**	3 (33,3%)				**9 (4%)	
**Arrhythmias**				20.3%		
**AF**	5 (55,6%)				44(21%)	1 (3%)
**NYHA >1**	26.4%				13 (35.1%)	13 (35.1%)
**Syncope**	8.3%					7 (19%)
**AVB II or III, sinus arrest, PM or ICD implantation**	4.2%				29 (14%)	
**CV death**	2				18 (9%)	
**Myocardial infarction**	0				22 (20%)	
**LVH, %**	16.6%		46.4%	55.4%	90/181 (50%)	21 (57%)
**LVMi (g/sqm)**	71.5 [57.2-86.2]		50.65±16.9 g/m	108±46 g/sqm		66±29
**EF, %**	65 [62-67.7]					64±10
**E/e′**	7 [6-10]					
**LAVi**	24 [17-31]					37±14
**TR-Vmax**	2.3 [1.9-2.4]					
**GLS, %**	-18 [-16/ -21]					-20±4%*
**MD, msec**	39 [29-60]					
**CMR done**	19.4%				141 (66%)	100%
**LGE**	12.5%				54/139 (39%)	16 (43%)
**ERT**	25%			57.6%	60%	20 (54%)
**Migalastat**	11.1%					
**untreated**	63.9%				40%	
**stroke**	8.3%			13.6%		
**CKD**	12.5%					

It should be noted that not all parameters were uniformly assessed across all studies. In light of this, only those parameters that were explicitly reported for each study have been included in the table for comparative purposes.

*CMR-evaluated parameters.

**SVA: composite of SCD, sudden cardiac arrest, sustained ventricular tachycardia, including appropriate ICD shock, and ventricular fibrillation.

For abbreviations: FOS: Fabry Outcome Survey ; for others see previous tables.

Table S12. Sex-based subgroup comparison, with a focus on female cohorts from hystorical registries and studies.

	Our female cohort N=53	FOS (Linhart 2006) [6] F=254	FOS, Hughes 2011 [7] F=78	FOS (Kampmann, 2015) [1] F=24	FOS (Ramaswami 2019), [2] F=34	FAMOUS (Lenders, 2020) [3], F=28	Avanesov 2023 [5], classical Females (N=20)	Spanish registry, (Sanchez, 2023) [8] F=97
**Age (yo)**	62,5 ± 3,9	35.8±18.7		38.6±14.2		51±11	43±12	50.1 ± 17.2
**Age ERT start**				38.7±14.1	46.70 (3.7–67.3)			
**Classic variant**	17%						51±15	39 (40.2%)
**Late onset**	43.4%							52 (53.6%)
**VUS**	39.6%							6 (6.2%)
**α-Gal A (nmol/ml/h)**	7.9 [4.6-11.3]							15 (51.7%) decreased
**Lyso Gb3 (nmol/l)**	1.5 [1.1-1.7]							2.4 [1-11]
**hypertension**	33.9%			8.3%		58.3%		
**diabetes**	16.1%			0		11.5%		
**BPsys**	120 [115-130]	120±16		127.3 ±13.2	123.7±17.4			
**BPdia**	75 [70-80]	71±12		72.3± 9.1	72.6±10.7			
**smoke**	19.6%	21.9%		8.3%		16.7%		
**SCD**	1.9%							1 (1.1%)
**Conduction abnormalities**	3.8%	6.7%	37.5%					
**PM/ICD**	0	1.6%				0/0		
**arrhythmias**	17%	21.3%	21.1%					
**V-AR**	7.5%							4 (4.4%)
**AF**	9.4%							2 (2.2%)
**LVH**	17%	21.3%	25.5% LVMi >48 g/m	67%	52.9%	39.3%		35 (38.5%)
**Reduced exercise**	32.1%	19.7%	33.3%	31%				9 (9.9%)

**tolerance (NYHA>1)**								
**syncope**	10.7%	2.4%						5 (5.5%)
**LVMi (g/sqm)**	66 [54-85.5]		48.2 ±17 (g/m)	MWT 11.7 ± 2.5 (8.7-17 mm)	52.2 ±16.99	85±19	72±8	
**EF, %**	65 [62-67.5]							
**E/e′**	8 [6-10]							
**GLS, %**	-19 [-17,-22]						-22±4%	
**MD, msec**	37 [29.2-49.7]							
**LGE at CMR**	57%						5 (25%)	2 (2.2%)
**stroke**	7.1%	4.1%				7.1%		3 (3.3%)
**CKD**	10.7%	0.8%						7 (7.7%)

The data is sourced both from extensive registries such as FOS and Famous, as well as studies that align closely with our own methodological framework. It is crucial to note that Avanesov's study relies on CMR imaging parameters, which somewhat constrains its comparability with other echo-based studies. Moreover, older studies utilized cardiac mass indexed in grams/meter rather than per square meter, which makes the numerical data less directly comparable. Recent years have seen the incorporation of advanced techniques derived from speckle tracking and MRI applications with LGE, that were conspicuously absent in older studies. For a nuanced comparison of our data, we have opted to include studies by El Sayed for the first table, and the Spanish registry along with Avanesov's MRI-based study for the second. Regarding MD, we have yet to find references in large, multicentric registries, making our study among the pioneering efforts to evaluate this particular parameter. For abbreviations: see previous tables.

References

1. Kampmann C, Perrin A, Beck M. Effectiveness of agalsidase alfa enzyme replacement in Fabry disease: Cardiac outcomes after 10 years' treatment. *Orphanet J. Rare Dis.* **2015**, *10*, 125.
2. Ramaswami U, Beck M, Hughes D, Kampmann C, Botha J, Pintos-Morell G, West ML, Niu DM, Nicholls K, Giugliani R; FOS Study Group. Cardio- Renal Outcomes With Long- Term Agalsidase Alfa Enzyme Replacement Therapy: A 10- Year Fabry Outcome Survey (FOS) Analysis. *Drug Des. Devel. Ther.* **2019**, *13*, 3705–3715.
3. Lenders M, Nordbeck P, Kurschat C, Karabul N, Kaufeld J, Hennermann JB, Patten M, Cybulla M, Müntze J, Üçeyler N et al. Treatment of Fabry's Disease With Migalastat: Outcome From a Prospective Observational Multicenter Study (FAMOUS). *Clin. Pharmacol. Ther.* **2020**, *108*, 326–337.
4. El Sayed M, Hirsch A, Boekholdt M, van Dussen L, Datema M, Hollak C, Langeveld M. Influence of sex and phenotype on cardiac outcomes in patients with Fabry disease. *Heart* **2021**, *107*, 1889–1897.
5. Avanesov M, Asgari A, Muschol N, Köhn AF, Tahir E, Adam G, Kirchhof P, Lund G, Cavus E, Patten M. Comparison of classical Fabry and its p.D313Y and p.A143T variants by cardiac T1 mapping, LGE and feature tracking myocardial strain. *Sci. Rep.* **2023**, *13*, 5809.
6. Linhart A, Kampmann C, Zamorano JL, Sunder-Plassmann G, Beck M, Mehta A, Elliott PM; European FOS Investigators. Cardiac manifestations of Anderson-Fabry disease: Results from the international Fabry outcome survey. *Eur. Heart J.* **2007**, *28*, 1228–1235.
7. Hughes DA, Barba Romero MÁ, Hollak CE, Giugliani R, Deegan PB. Response of women with Fabry disease to enzyme replacement therapy: Comparison with men, using data from FOS--the Fabry Outcome Survey. *Mol. Genet. Metab.* **2011**, *103*, 207–214.
8. Sánchez R, Ripoll-Vera T, López-Mendoza M, de Juan-Ribera J, Gimeno JR, Hermida Á, Ruz-Zafra MA, Torregrosa JV, Mora A, García-Pinilla JMet al. The Spanish Fabry women study: A retrospective observational study describing the phenotype of females with GLA variants. *Orphanet J. Rare Dis.* **2023**, *18*, 8.
